# Noncoding Variants in Intron 2 of *HK1* Associated With Hyperinsulinism With Variable Clinical Phenotype

**DOI:** 10.1210/clinem/dgag108

**Published:** 2026-03-10

**Authors:** Kara E Boodhansingh, Katherine Lord, Winnie Sigal, Joshua E Benjet, Pan Chen, Christine A Juliana, Lauren Mitteer, Tricia Bhatti, Charles A Stanley, Diva D De Leon, Arupa Ganguly

**Affiliations:** Congenital Hyperinsulinism Center, Division of Endocrinology and Diabetes, Children's Hospital of Philadelphia, Philadelphia, PA 19104, USA; Congenital Hyperinsulinism Center, Division of Endocrinology and Diabetes, Children's Hospital of Philadelphia, Philadelphia, PA 19104, USA; Department of Pediatrics, Perelman School of Medicine at the University of Pennsylvania, Philadelphia, PA 19104, USA; Congenital Hyperinsulinism Center, Division of Endocrinology and Diabetes, Children's Hospital of Philadelphia, Philadelphia, PA 19104, USA; Department of Pediatrics, Perelman School of Medicine at the University of Pennsylvania, Philadelphia, PA 19104, USA; Congenital Hyperinsulinism Center, Division of Endocrinology and Diabetes, Children's Hospital of Philadelphia, Philadelphia, PA 19104, USA; Congenital Hyperinsulinism Center, Division of Endocrinology and Diabetes, Children's Hospital of Philadelphia, Philadelphia, PA 19104, USA; Congenital Hyperinsulinism Center, Division of Endocrinology and Diabetes, Children's Hospital of Philadelphia, Philadelphia, PA 19104, USA; Congenital Hyperinsulinism Center, Division of Endocrinology and Diabetes, Children's Hospital of Philadelphia, Philadelphia, PA 19104, USA; Department of Pathology, Children's Hospital of Philadelphia, Philadelphia, PA 19104, USA; Department of Pathology, Perelman School of Medicine at the University of Pennsylvania, Philadelphia, PA 19104, USA; Congenital Hyperinsulinism Center, Division of Endocrinology and Diabetes, Children's Hospital of Philadelphia, Philadelphia, PA 19104, USA; Department of Pediatrics, Perelman School of Medicine at the University of Pennsylvania, Philadelphia, PA 19104, USA; Congenital Hyperinsulinism Center, Division of Endocrinology and Diabetes, Children's Hospital of Philadelphia, Philadelphia, PA 19104, USA; Department of Pediatrics, Perelman School of Medicine at the University of Pennsylvania, Philadelphia, PA 19104, USA; Department of Human Genetics, Perelman School of Medicine at the University of Pennsylvania, Philadelphia, PA 19104, USA

**Keywords:** pancreas, insulin, hypoglycemia, beta cells

## Abstract

**Context:**

Noncoding variants in hexokinase 1 (*HK1*) were first associated with congenital hyperinsulinism (HI) in a large family with diazoxide-responsive HI in 2008. Since then, additional cases have been reported in the literature with noncoding variants in *HK1* associated with variable HI phenotypes.

**Methods:**

We sequenced a 350 bp region in intron 2 of *HK1* in 281 individuals with genetics-negative HI to identify additional cases related to non-coding *HK1* variants and to characterize the clinical features of these cases.

**Results:**

We identified 16 unique non-coding variants in intron 2 of *HK1* in 18 individuals with genetics-negative HI (18/281, 6.4%). In 7 cases (7/18, 39%), the *HK1* variant was inherited from a parent (2 maternal, 5 paternal); 2 are known to be affected with HI. In 9 cases, the *HK1* variant was de novo (9/18, 50%). The age of presentation of HI ranged from day of life 1 to 21 months of age. Seven cases had diazoxide-responsive HI (7/18, 39%). Eleven cases were diazoxide unresponsive (11/18, 61%); 5 underwent pancreatectomy at ages ranging from 6 months to 3 years of age.

**Conclusions:**

Noncoding variants in intron 2 of the *HK1* gene have now been associated with HI in a growing number of cases. Our findings suggest that a significant proportion of individuals with negative genetics in genes currently known to be associated with HI may harbor *HK1* intron 2 variants. Identifying these cases is important for clinical care as well as for assessing recurrence risk for families.

Congenital hyperinsulinism (HI) is the most common cause of persistent hypoglycemia in infants and children and has been associated with mutations in a growing number of genes responsible for regulating insulin secretion (1). Andrew McQuarrie was the first to use the term “idiopathic hypoglycemia of infancy” in 1954 to classify patients with what we now refer to as HI (2). He described a sibling pair with hypoglycemia presenting in early infancy and treated with pancreatectomy without resolution of their hypoglycemia. Our group had the opportunity to study the extended family of this sibling pair originally described by McQuarrie. At that time, the family included 25 affected individuals spanning 4 generations with a dominant form of HI that was well controlled with diazoxide and mapped to chromosome 10q by linkage analysis (3). Within the chromosome 10q region, 3 noncoding variants in hexokinase 1 (*HK1*), including 1 within intron 2 (chr10:g.71108666dupG), were identified through whole-genome sequencing in affected individuals from this family. Since that report, additional cases of HI have been associated with noncoding sequence variants, as well as large deletions, in intron 2 of *HK1* (4, 5).

In pancreatic β-cells, glucose phosphorylation is normally carried out by glucokinase (GCK), a hexokinase with low affinity for glucose that is responsible for setting the β-cell threshold for insulin release at concentrations of ∼5 mM (6-8). Hexokinase 1, encoded by the *HK1* gene, is a glycolytic enzyme that has a higher affinity for glucose compared to glucokinase. Though widely expressed across human tissues, *HK1* is not expressed in pancreatic β-cells, thus preventing insulin secretion at low plasma glucose concentrations. We and others have postulated that variants in noncoding regions of *HK1* might disrupt the normal silencing of the gene in β-cells, thereby resulting in aberrant *HK1* expression and hypoglycemia (3, 4).

This study aimed to identify individuals with HI who have noncoding variants in intron 2 of *HK1* among those who had not been found to have coding sequence mutations in known HI-associated genes. We describe the molecular and clinical features of these cases here.

## Materials and Methods

### Probands

Individuals were included in this study if they had (1) biochemically confirmed HI based on previously described criteria (9) and (2) negative coding sequence variant analysis for genes associated with HI in peripheral blood DNA. Mutation analysis of genomic DNA was performed in commercial laboratories using a 2-tier approach. Tier 1 included direct sequencing of the 3 most common HI genes (*ABCC8*, *KCNJ11*, and *GCK*); reflexive tier 2 testing was then performed by next-generation sequencing for *ABCC8*, *KCNJ11*, *GCK*, *GLUD1*, *UCP2*, *HNF1A*, *HNF4A*, *HADH*, and *SLC16A1*. In some cases, *INSR, AKT2, CACNA1D, FOXA2, KDM6A, KMT2D, PGM1, PMM2*, and *TRMT10A* were also included in reflexive tier 2 testing.

Written informed consent was provided by parents/guardians of all participants, and assent was obtained, as appropriate. These studies were approved by the Children's Hospital of Philadelphia (CHOP) Committees for the Protection of Human Subjects (IRB 07-005772 and 08-006216).

### Genetic Analysis

Genomic DNA was isolated from peripheral blood (5 Prime Sciences, Gaithersburg, MD, USA) or from saliva (Oragene^TM^ DNA, DNA Genotek, Kanata, Ontario, Canada). DNA from surgical pancreatic specimens (whole pancreas or isolated islets) was extracted using the DNA/RNA AllPrep kit (QIAGEN, Valencia, CA, USA).

Direct sequencing for a 350 bp region encompassing chr10:71108585-71108933 in intron 2 of *HK1* was performed under a research protocol as previously described (10). In 1 patient (case 16), the intron 2 region was analyzed for a possible large deletion using long-range PCR (Takara Bio, Inc., San Jose, CA, USA) followed by gel extraction and direct sequencing of the resulting fragments. Variant screening, including deletion/duplication analysis for *HK1* intron 2, was done commercially in cases 10, 17, and 18.

*HK1* variants are listed according to their genomic positions on chromosome 10 (GRCh37/hg19). Variants were searched against the Genome Aggregation Database Browser, version 2.1 (gnomAD) (11).

### *HK1* Expression by Immunoflourescence and cDNA Analysis

#### Immunofluorescence

Paraffin-embedded formalin-fixed pancreatic tissue obtained at the time of surgery was sectioned and mounted onto glass slides. Paraffin was removed with xylene followed by rehydration with ethanol. Antigen retrieval was done with sodium citrate buffer. Slides were incubated with primary antibodies [rabbit anti-HK1 1:250 (RRID: AB_2117001), and goat anti-insulin 1:500 (RRID: AB_2296108), Santa Cruz Biotechnology, Dallas, TX, USA] overnight at 4 °C followed by incubation with the secondary antibodies [488 donkey anti-goat 1:500 (RRID: AB_2762833), Cy3 donkey anti-goat 1:500 (RRID: AB_2762840), and 4′,6-diamidino-2-phenylindole 1:1000] for 1 hour in a hydration chamber at room temperature. Slides were scanned into ImageScope Software (Leica Biosystems, Deer Park, IL, USA) and analyzed using the Indica Cyto-Nuclear FL v1.3 algorithm (Indica Labs, Albuquerque, NM, USA). β-cells were identified based on positive staining for insulin. β-cells positive for HK1 staining were counted against β-cells negative for HK1 staining, and cytoplasmic intensity of HK1 staining per islet was calculated. Age-matched control samples were obtained from leftover surgical specimens.

#### cDNA analysis of *HK1* expression

RT-PCR of cDNA was performed using custom probes for the ubiquitous *HK1* isoform (sequences available upon request); glyceraldehyde-3-phosphate dehydrogenase was used as the endogenous control. Reactions were prepared in triplicate for each sample using PrimeTime Gene Expression Master Mix (Integrated DNA Technologies, Coralville, IA, USA) and run on a Quant Studio 6 (Thermo Fisher Scientific, Waltham, MA, USA) using standard cycling. Analysis was performed using the delta-delta computed tomography method using a commercially available pancreatic RNA sample as the calibrator (Life Technologies, Carlsbad, CA, USA).

## Results

Mutation screening for variants in a 350 bp region in intron 2 of *HK1* was completed in genomic DNA from blood or saliva in 281 individuals with HI seen at the CHOP Congenital Hyperinsulinism Center between 1997 and 2024 and in whom a pathogenic sequence variant in genes known to cause HI had not been identified.

### *HK1* Intron 2 Variants

As shown in Table 1, variants in intron 2 of *HK1* were identified in 18 of the 281 individuals screened (18/281, 6.4%). Sixteen unique variants were identified in these 18 individuals.

**Table 1:** Clinical characteristics of 18 probands with HI and intron 2 *HK1* variants

Case ID	*HK1* variant (inheritance)	Birth weight (g)	Z-score	Age of presentation	Pancreatectomy age (% resected, histology)	Current age (years)	Last known treatment (age)
1	10:71108644a>t (de novo)	3710	0.75	DOL 2 (shaking)	11 mos (2%, diffuse)	8	Diazoxide (2 years)
2	10:71108644a>t (de novo)	2721	1.15	DOL 2	—	18	Diazoxide (18 years)
3	10:71108644a>c (paternal)	4320	2.94	DOL 1	—	7	Diazoxide + lanreotide (1 year)
4	10:71108652a>g^*a*^ (maternal)	2780	−1.76	4 mos (seizure)	9 mos (95%, diffuse)	22	Diabetes (12 years)
5	10:71108652a>t (de novo)	3430	0.03	DOL 2 (seizure)	3 yrs (95%, diffuse)	17	Diabetes (16 years)
6	10:71108652a>g^*a*^ (maternal)	3231	−0.38	4 mos (abnormal eye movements)	—	34	Octreotide (33 years)
7	10:71108652-71108671del (de novo)	4678	2.21	DOL 1	—	21	Diazoxide (20 years)
8	10:71108653c>t*^a^* (de novo)	3949	0.33	3 mos	—	23	Limited Fasting (9 years)
9	10:71108660g>t (de novo)	3288	−0.26	4 mos (seizure)	14 mos (2%, diffuse)	13	Diazoxide (12 years)
10	chr10:71108665c>g^*b*^ (paternal)	3628	0.41	11 mos (lethargy)	—	2	Diazoxide (2 years)
11	chr10:71108666dupG^c^ (paternal)	3405	−0.35	3 mos (seizure)	—	23	Diazoxide (11 years)
12	10:71108688g>t (de novo)	3125	−0.07	21 mos (seizure)	—	21	Diazoxide (20 years)
13	10:71108688_71108692del (de novo)	4900	2.62	DOL 1	—	9	Diazoxide + lanreotide (9 years)
14	10:71108710t>c (paternal)	3721	0.24	DOL 28 (seizure)	6 mos (98%, diffuse)	14	Diabetes (12 years)
15	10:71108795g>a (paternal)	3345	−0.48	4 mos (seizure)	—	4	Diazoxide (3 years)
16	10:71104142-71107810del (unknown)	n/a	n/a	DOL 1	—	37	Diazoxide + lanreotide (34 years)
17	10:71106798_71111753del (de novo)	3945	1.23	DOL 1	—	10	Diazoxide + lanreotide (10 years)
18	10:71108294_71117480del (unknown)	4950	2.71	DOL 1	—	2	Diazoxide + lanreotide (2 years)

Abbreviations: DOL, day of life; HI, hyperinsulinism; *HKI*, hexokinase 1; mos, months.

^*a*^PMID 40033430.

^*b*^PMID 36333503.

^*c*^PMID 23859901.

**Figure 1:**
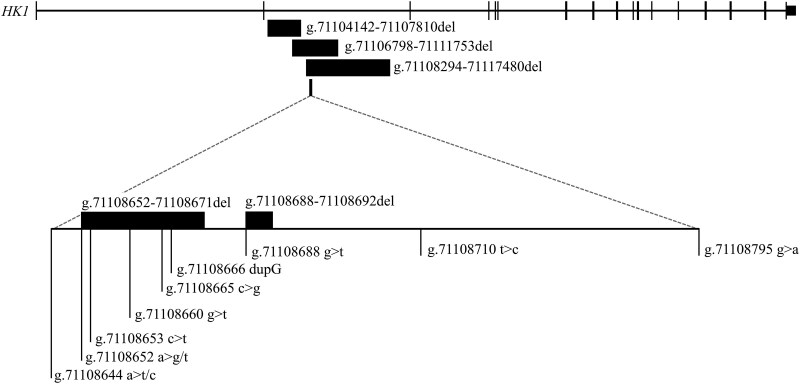
Location of *HK1* intron 2 variants identified in 18 cases at CHOP. Shown are the 17 exons and intronic regions of the ubiquitously expressed isoform of *HK1* (NM_000188.3). Large deletions are shown below the gene diagram. An expanded view of a 151 bp region, which includes 2 small deletions and 11 single nucleotide variants, is shown at the bottom of the figure. Genomic coordinates corresponding to hg19 are used to indicate variant location. Abbreviation: CHOP, Children's Hospital of Philadelphia.

Eleven of the variants are single nucleotide substitutions, 2 are small deletions of 4-19 nucleotides, and 3 are large deletions ranging from 3668 to 9186 base pairs within intron 2 of *HK1* (Fig. 1). Four of these variants have been reported in the literature, including the variant identified in case 11, who is part of the large family from which McQuarrie originally described idiopathic hypoglycemia of infancy in a sibling pair, and reported by our group previously (Table 1). All 16 variants are absent from large control population databases (gnomAD).

In 9 cases, the intron 2 *HK1* variant appeared to have arisen de novo, although nonpaternity was not excluded (9/18, 50%). Seven cases (7/18, 39%) inherited the variant from a parent. Two of these parents were known to have HI, both with a history of pancreatectomy (cases 3, 14). The carrier parent of case 10 underwent a 24-hour fast as part of a research study, which showed evidence of hyperinsulinism. The father of case 11 did not report symptoms of hypoglycemia, although he is an obligate carrier and is part of the large family with 25 affected individuals who also carry the *HK1* intron 2 variant (3). The remaining carrier parents had no noted symptoms of HI (cases 4, 6, 15). Inheritance could not be established in the remaining 2 cases.

### Clinical Features

As shown in Table 1, most of the cases described here had an appropriate for gestational age birth weight (13/17, 76%); 4 of the cases were born large for gestational age (4/17, 24%). Nine cases presented with hypoglycemia within the first days of life (9/18, 50%), 8 presented within the first year of life (8/18, 44%), and only 1 presented at over 1 year of age (1/18, 6%). In the 9 cases where presenting symptoms were reported, seizure or seizure-like activity was the most common symptom exhibited (8/9 cases, 88.9%); lethargy was reported in the remaining case.

Three of the 18 cases underwent near-total pancreatectomies at ages ranging from 6 months to 3 years of age (cases 4, 5, and 14). All of these cases developed postpancreatectomy diabetes by 12 years of age. An additional 2 cases had biopsies of their pancreas without resection. Histology of pancreatic parenchyma in the biopsies and resection specimens showed overall intact lobular architecture with variable numbers of islet cells demonstrating nuclear enlargement ranging from rare to more conspicuous, most consistent with diffuse disease. In cases where p57 staining was performed at the time of diagnosis, nuclear immunoreactivity was retained.

Of the 15 cases that did not undergo near-total pancreatectomy, hypoglycemia was treated with diazoxide alone in 9 individuals (9/15, 60%) and with octreotide alone in 1 individual (1/15, 7%). One case that was treated with diazoxide alone was able to discontinue therapy at 9 years of age (case 8). The remaining five cases required a combination of diazoxide plus lanreotide to control hypoglycemia (5/15, 33%).

### *HK1* Expression by Immunoflourescence and cDNA Analyses

**Figure 2:**
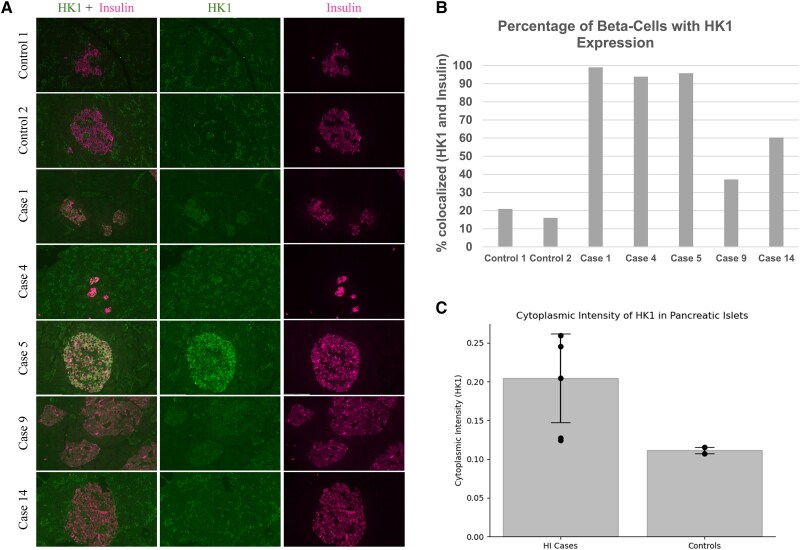
Colocalization of HK1 and insulin expression by immunofluorescence staining. Formalin-fixed paraffin-embedded tissue sections from 5 cases of HI with *HK1* intron 2 variants compared to 2 non-HI age-matched controls. (A) Immunofluorescence images from cases and controls. Insulin-staining β-cells are shown in pink and HK1 staining in green. The first column shows both stains together while staining for HK1 only is shown in the second column, and staining for insulin only is shown in the third column. (B) The proportion of β-cells positive for HK1 staining is higher in all 5 cases compared to control tissue. (C) The average cytoplasmic intensity of HK1 staining in pancreatic islets is higher in HI cases compared to controls. Bars show the median of the average cytoplasmic intensity of cases or controls, while individual data points refer to the average cytoplasmic intensity in pancreatic islets from each case or control. Error bars indicate SD. The *P*-value between cases and controls is .47. Abbreviations: HI, hyperinsulinism; HKI, hexokinase 1.

To evaluate whether variants in intron 2 of *HK1* resulted in expression of *HK1* in the β-cells, we performed immunofluorescent staining for HK1 in formalin-fixed paraffin-embedded sections of the pancreas from the 3 cases who had near-total pancreatectomy (cases 4, 5, 14) and the 2 cases where biopsies only occurred (cases 1, 9). These findings were compared to the pancreas from age-matched controls without HI. Pancreas sections were stained with insulin to identify pancreatic β-cells, as well as HK1 (Fig. 2A). As shown in Fig. 2B, the proportion of β-cells positive for HK1 staining was higher in all 5 cases compared to control tissue. In 3 of the cases (cases 1, 4, and 5), nearly all of the β-cells also had increased HK1 expression, while the proportion of β-cells expressing HK1 in cases 9 and 14 was lower (∼40-60%). In addition, the average cytoplasmic intensity of HK1 staining in islets was higher in cases compared to controls (Fig. 2C).

**Figure 3:**
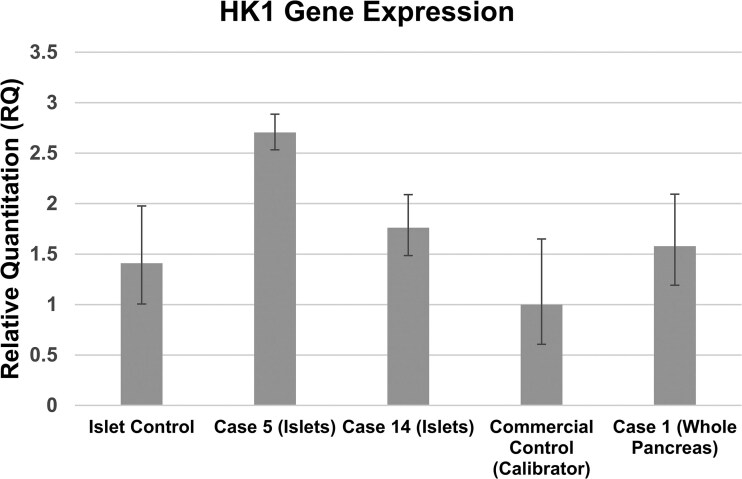
*HK1* expression by RT-PCR. *HK1* expression was assessed by RT-PCR in 3 cases of HI with *HK1* intron 2 variants compared to non-HI control pancreas. One case (case 5) showed a modest increase in *HK1* expression compared to control (∼1.5-fold increase). Bars represent average RQ across 3 technical replicates, and error bars show the maximum and minimum RQ. Abbreviations: HI, hyperinsulinism; *HKI*, hexokinase 1; RQ, relative quantitation.

To determine whether the increase in HK1 immunofluorescence reflected increased *HK1* gene expression, we measured pancreatic tissue levels of *HK1* mRNA by synthesizing cDNA by RT-PCR in those cases from which fresh or frozen whole pancreas (case 1) or islets (cases 5, 14) were available. As shown in Fig. 3, only case 5 showed a mild increase in *HK1* gene expression compared to control islets (∼1.5-fold increase).

## Select Case Summaries

Case 6 is a 34-year-old female who was born full term with a gestationally age-appropriate birth weight of 3.231 kg (*z*-score −0.38). Her neonatal period was unremarkable. At 4 months of age, her mother noticed abnormal eye movements, and she was found seizing after sleeping overnight. She was diagnosed with HI and treated with diazoxide until 5 years of age. She reportedly did not experience symptomatic hypoglycemia after discontinuing diazoxide until the age of 22, at which point she started having intermittent symptoms that increased in frequency and severity over a 6-year period. At the age of 28, she underwent a diagnostic evaluation with a critical sample significant for a plasma glucose of 40 mg/dL (2.2 mmol/L), elevated insulin of 76 uIU/mL (528 pmol/L), and C-peptide of 7.75 ng/mL (2.6 nmol/L). Insulin autoantibodies were negative, and IGF-I and IGF-II were normal. She was started on diazoxide but continued to experience episodes of symptomatic hypoglycemia occurring both during the day and with fasting.

At 28 years of age, she was still experiencing hypoglycemia while on diazoxide and was seen at CHOP for a second opinion. She was initiated on lanreotide, and her diazoxide was subsequently discontinued. She underwent a fasting tolerance test where she demonstrated the ability to fast for 18 hours with normal plasma glucoses. She completed a mixed-meal tolerance test and oral protein tolerance test while on treatment, both of which showed mild hypoglycemia, suggesting that she has mild protein-induced hypoglycemia. She was also on metformin for polycystic ovarian syndrome. At home, she continued to experience hypoglycemia while on lanreotide with no clear correlations or contributing factors. The frequency of these episodes increased over time. At 31 years of age, lanreotide was discontinued, and she was started on octreotide in preparation for a potential pregnancy. She continues to experience hypoglycemia periodically while on octreotide.

Genetic testing for genes known to be associated with HI (*ABCC8, KCNJ11, GCK, GLUD1, HADH, UCP2, HNF4A, HNF1A, SLCA61A1, INSR, AKT2, CACNA1D, FOXA2, KDM6A, KMT2D, PGM1, PMM2*, and *TRMT10A*) was done commercially and was negative. She was found to have a variant in intron 2 of *HK1* (10:71108652a>g), inherited from her mother. Her mother denied symptoms of hypoglycemia. This variant has been previously reported in the literature associated with HI (4).

Case 17 is a 9-year-old male who was born full term with a birth weight of 3.945 kg (*z*-score 1.23). He was found to have hypoglycemia at birth. He was diagnosed with HI at an outside hospital and started on diazoxide at the maximum dose of 15 mg/kg/day. Genetic testing for genes known to be associated with HI (*ABCC8, KCNJ11, GCK, GLUD1, HADH, UCP2, HNF4A, HNF1A, SLCA61A1*, and *INSR*) was done commercially and was negative. Methylation analysis for Beckwith–Weidemann syndrome was also negative. Whole-exome sequencing did not reveal variants that could explain his HI. At 5 months of age, he was noted to have multiple episodes of prefeed hypoglycemia while on diazoxide, and he was transferred to CHOP for further evaluation. At CHOP, he underwent a fasting test on the maximum dose of diazoxide (15 mg/kg/day) to evaluate diazoxide responsiveness. He fasted for a total of 14.5 hours, maintaining plasma glucoses above 70 mg/dL (3.9 mmol/L) for only 3 hours. At the end of the fast, his plasma glucose was 46 mg/dL (2.6 mmol/L) with a betahydroxybutyrate of 17.7 mg/dL (1.7 mmol/L), which was consistent with a partial response to diazoxide. His diazoxide was discontinued, and he underwent an 18F-fluorodihydroxyphenylalanine positron emission tomography scan, which did not identify a focal lesion. Off diazoxide, he required an IV glucose infusion rate (GIR) of 15 mg/kg/min to maintain euglycemia. Given the high GIR requirement, diazoxide was restarted at the maximum dose (15 mg/kg/day), but he still required enteral and IV dextrose to maintain plasma glucose levels above 70 mg/dL (3.9 mmol/L). As such, he was initiated on octreotide therapy. His final regimen at discharge included octreotide during the day (8 mcg/kg/day) with a continuous intragastric infusion of dextrose 20% overnight (GIR 5.2 mg/kg/min) and diazoxide every 12 hours (15 mg/kg/day). Lanreotide was initiated at 13 months of age, at which point both octreotide and dextrose were discontinued. His diazoxide dose was subsequently decreased to 11.8 mg/kg/day, and his glucoses were stable. At the time of publication, he was 9 years of age and has remained on lanreotide and diazoxide. His diazoxide dose has been adjusted over this time period in response to ongoing hypo- and hyperglycemia. He has also required enteral dextrose periodically. His plasma glucoses have continued to be difficult to manage, with frequent intermittent episodes of symptomatic hypoglycemia necessitating visits to the emergency department. He also has required tube feeds due to feeding issues.

He was subsequently found to have a large deletion in intron 2 of *HK1* (chr10:71106798_71111753del) that was found to be de novo.

## Discussion

We identified noncoding variants in a 350 bp segment of the intron 2 region of *HK1* in 18 of 281 individuals with genetics-negative HI. The results of these studies demonstrate that noncoding variants in intron 2 of *HK1* account for a significant proportion of genetics-negative HI cases with a minimum prevalence of 6.4% of unsolved cases in our cohort (18/281). These results are similar to previous reports of HK1-HI from Wakeling et al (9%), Bennett et al (5%), and Velde et al (3%) (4, 5, 12). Other well-established genetic forms of HI, including *HNF4A* and *HNF1A*, are reported to have a similar prevalence (10).

This report expands the total number of variants reported in intron 2 of *HK1* to 44. With the exception of the large deletion identified in case 16 of the present report, the remainder of the variants share a minimal region of overlap encompassing 153 base pairs (Chr10:71108642-71108795), expanding the minimal regulatory region previously reported by Bennett et al by 111 bp (5). Because this region is large and expanding, it is possible that there are multiple transcription control regions in this intron, which may account for some of the phenotypic variability seen within patients. While functional studies are needed to definitively establish these variants as pathogenic, there is strong evidence of pathogenicity for the variants included here based on the American College of Medical Genetics and Genomics guidelines (13). In the current series, the mutation arose de novo in 8 cases and was inherited from a parent in 7 cases. Of the 7 carrier parents, 2 have a clear history of HI requiring pancreatectomy, 1 is an obligate carrier from the large family previously reported by our group, and 1 has evidence of hyperinsulinism in response to a 24-hour fast. None of these variants is present in control populations (gnomAD). Four of the variants have been previously reported to be associated with HI, and all are located within a small region of *HK1* intron 2 (Chr10:71108642-71108795) in which 29 other variants have been associated with HI (3-5).

A limitation of the present study was that the analysis in intron 2 of *HK1* included only a 350 bp region and did not include deletion/duplication testing in most patients. It is possible that by expanding the region of analysis and including deletion/duplication testing in the cases that did not have a variant within the 350 bp sequenced region would yield additional HK1-HI cases and help to clarify the minimal regulatory region in *HK1* intron 2.

Noncoding intron 2 variants in *HK1* were first associated with HI by our group in a large family with a dominant mode of inheritance in which affected individuals were well controlled on diazoxide (3). In subsequent reports, including the patients presented here, the phenotype associated with HK1-HI has been more variable. As illustrated by the cases presented here, diazoxide is found to be effective in less than 50% of cases (8/18, 44%). In cases of diazoxide-unresponsive HI, some cases have been treated with a combination of diazoxide plus somatostatin analogs such as octreotide or lanreotide, while others have undergone pancreatectomy in an effort to control hypoglycemia. Postoperatively, all 3 cases in the current series that underwent near-total pancreatectomy continued to have hypoglycemia immediately following surgery, requiring continued medical management that eventually transitioned to diabetes. Clinically, the poor response to diazoxide seen in cases of HK1-HI, as well as the presence of persistent hypoglycemia following pancreatectomy, is similar to what is observed in children with activating mutations in glucokinase (*GCK*). Given the limited success of pancreatectomy in controlling hypoglycemia, it has become the practice at our institution to not pursue surgery in these cases as it is often not therapeutically successful and may result in diabetes as the patients get older. It is therefore important that mutation analysis include the intron 2 region of *HK1* in cases of diazoxide-unresponsive HI prior to considering pancreatectomy.

The HI associated with *HK1* intron 2 variants persists into adulthood and can vary in severity throughout the lifespan. In the current series, the oldest individual is 37 years of age and continues to require treatment to control hypoglycemia. As described in the Selected Case Summaries, case 6 was treated with diazoxide in childhood and experienced a period of time between the ages of 5 and 22 years of age without hypoglycemia symptoms prior to again requiring therapy for hypoglycemia that continues into her 30s. Additionally, in the large family with HK1-HI described by Pinney et al, the grandmother of case 11 described in the present report, who was an obligate carrier but not known to have hypoglycemia for most of her life, suffered a hypoglycemic seizure at the age of 89 years following a 10-pound weight loss from adult-onset celiac disease (3). This highlights the importance of continued evaluation for hypoglycemia and appropriate medical management in patients with HK1-HI as well as for their family members who are found to also carry the intron 2 *HK1* variant. Hypoglycemia symptoms were denied by the carrier parents in 3 of the 7 cases presented here with an inherited intron 2 *HK1* variant (3/7, 43%). In other dominant forms of HI, such as HI caused by inactivating mutations in the genes encoding the subunits of the β-cell adenosine triphosphate-dependent potassium channel (K_ATP_-HI), we have observed a similar proportion of parents who carry a dominant mutation and deny symptoms of hypoglycemia (14). In these cases of K_ATP_-HI adult carriers, hypoglycemia is often unmasked following provocative testing such as a 24-hour fasting test or oral protein tolerance test. It is currently unknown but seems likely that provocative testing would yield similar results in adult carriers of intron 2 variants in *HK1*.

It has been speculated that noncoding variants in intron 2 of *HK1* result in HI due to aberrant expression of HK1 in β-cells due to loss of repression. Increased HK1 expression has previously been reported in the pancreas from some children with HI and histology classified as localized islet nuclear enlargement (LINE-HI) (15). Henquin et al demonstrated HK1 expression in hyperfunctional β-cells from children in 5 cases of HI with “atypical”/LINE-HI pathology and negative genetic analysis by immunofluorescence (16). In 2 of these cases, mutation analysis for *GCK* was negative in DNA isolated from hyperfunctional β-cells (*HK1* mutation analysis was not done). We have also demonstrated increased HK1 expression by immunofluorescence and RT-PCR in abnormal pancreas from 2 children with LINE-HI pathology who had negative genetic testing for HI-related genes, including intron 2 of *HK1* in blood and the pancreas (15). In 1 additional case with LINE-HI, HK1 expression was increased by RT-PCR but not by immunofluorescence. The underlying mechanism for this increased HK1 expression in some cases of LINE-HI is not known. In addition to those with LINE-HI, increased HK1 expression has also been reported in other forms of insulin dysregulation, including those with K_ATP_-HI and type 2 diabetes (17, 18).

Wakeling et al previously reported an increase in HK1 protein expression based on immunostaining in islets of affected pancreas in 2 cases with noncoding *HK1* intron 2 variants (4). In the current series, both an increase in the proportion of β-cells with detectable HK1 protein immunostaining as well as an increase in the level of HK1 expression as determined by HK1 cytoplasmic intensity was demonstrated in all 5 cases from which pancreas was available for testing, though there is some variability between cases. However, only a mild increase in *HK1* expression based on RT-PCR was observed in 1 of 3 cases. It is possible that in the RT-PCR data, HK1 mRNA levels from non-β-cells could have masked any possible increase specific to the β-cells that is observed at the protein level by the immunofluorescence. Other possible causes for this discrepancy are that the *HK1* variants are creating an alternate transcript or that the transcripts are undergoing posttranscriptional modification, both of which may not be detectable using our RT-PCR assay. Because the tissue samples were collected at different time points, it is also possible that the disparity in HK1 expression levels is due in part to variations in tissue collection and fixation between cases.

In conclusion, HK1-HI due to noncoding variants in intron 2 of the *HK1* gene appears to account for an important proportion of HI cases and should be included on standard clinical genetic testing panels for HI. Insulin-mediated fetal overgrowth is not a consistent feature of this form of HI in that most children are born with appropriate for gestational age birth weights. These children often present with hypoglycemia within the first days of life, but later ages of presentation are also observed. While some children with intron 2 variants in *HK1* are not well controlled on diazoxide, pancreatectomy may not be effective in controlling hypoglycemia in these cases. The HI in these patients persists into adulthood and should be closely monitored throughout life. The evidence of increased HK1 expression in the pancreas from some cases of HK1-HI supports the hypothesis that these noncoding variants in *HK1* prevent the normal silencing of *HK1* in β-cells. However, the underlying mechanism responsible for the aberrant HK1 expression in these cases with *HK1* intron 2 variants remains to be elucidated.

## Acknowledgments

The authors would like to thank the Genome Aggregation Database and the groups that provided exome and genome variant data to this resource. A full list of contributing groups can be found at https://gnomad.broadinstitute.org/about.

## Data Availability

Some or all datasets generated during and/or analyzed during the current study are not publicly available but are available from the corresponding author on reasonable request.
